# Nicotinamide as potential biomarker for Alzheimer’s disease: A translational study based on metabolomics

**DOI:** 10.3389/fmolb.2022.1067296

**Published:** 2023-01-06

**Authors:** María C. Dalmasso, Martín Arán, Pablo Galeano, Silvina Perin, Patrick Giavalisco, Pamela V. Martino Adami, Gisela V. Novack, Eduardo M. Castaño, A. Claudio Cuello, Martin Scherer, Wolfgang Maier, Michael Wagner, Steffi Riedel-Heller, Alfredo Ramirez, Laura Morelli

**Affiliations:** ^1^ Laboratory of Brain Aging and Neurodegeneration-Fundación Instituto Leloir-IIBBA-National Scientific and Technical Research Council (CONICET). Ciudad Autónoma de Buenos Aires, Buenos Aires, Argentina; ^2^ Division of Neurogenetics and Molecular Psychiatry, Department of Psychiatry and Psychotherapy, Faculty of Medicine and University Hospital Cologne, University of Cologne, Cologne, Germany; ^3^ Studies in Neuroscience and Complex Systems Unit (ENyS-CONICET-HEC-UNAJ). Florencio Varela, Florencio Varela, Argentina; ^4^ Laboratory of NMR-Fundación Instituto Leloir-IIBBA-National Scientific and Technical Research Council (CONICET). Ciudad Autónoma de Buenos Aires, Cologne, Argentina; ^5^ Max Planck Institute for Biology of Ageing, Cologne, Germany; ^6^ Department of Pharmacology and Therapeutics, McGill University, Montreal, CA, Canada; ^7^ Department of Primary Medical Care, University Medical Centre Hamburg-Eppendorf, Hamburg, Germany; ^8^ Department of Neurodegenerative and Geriatric Psychiatry, University Hospital Bonn, Medical Faculty, Bonn, Germany; ^9^ German Center for Neurodegenerative Diseases (DZNE), Bonn, Germany; ^10^ Institute of Social Medicine, Occupational Health and Public Health, University of Leipzig, Leipzig, Germany; ^11^ Department of Psychiatry and Glenn Biggs Institute for Alzheimer’s and Neurodegenerative Diseases, San Antonio, TX, United States; ^12^ Cluster of Excellence Cellular Stress Responses in Aging-associated Diseases (CECAD), University of Cologne, Cologne, Germany

**Keywords:** NAD salvage pathway, vit B3, transgenic rats, alzheimer’s disease, biomarkers, brain alterations, nicotinamide (NAM), case-control analysis

## Abstract

**Introduction:** The metabolic routes altered in Alzheimer's disease (AD) brain are poorly understood. As the metabolic pathways are evolutionarily conserved, the metabolic profiles carried out in animal models of AD could be directly translated into human studies.

**Methods:** We performed untargeted Nuclear Magnetic Resonance metabolomics in hippocampus of McGill-R-Thy1-APP transgenic (Tg) rats, a model of AD-like cerebral amyloidosis and the translational potential of these findings was assessed by targeted Gas Chromatography-Electron Impact-Mass Spectrometry in plasma of participants in the German longitudinal cohort AgeCoDe.

**Results:** In rat hippocampus 26 metabolites were identified. Of these 26 metabolites, nine showed differences between rat genotypes that were nominally significant. Two of them presented partial least square-discriminant analysis (PLS-DA) loadings with the larger absolute weights and the highest Variable Importance in Projection (VIP) scores and were specifically assigned to nicotinamide adenine dinucleotide (NAD) and nicotinamide (Nam). NAD levels were significantly decreased in Tg rat brains as compared to controls. In agreement with these results, plasma of AD patients showed significantly reduced levels of Nam in respect to cognitively normal participants. In addition, high plasma levels of Nam showed a 27% risk reduction of progressing to AD dementia within the following 2.5 years, this hazard ratio is lost afterwards.

**Discussion:** To our knowledge, this is the first report showing that a decrease of Nam plasma levels is observed couple of years before conversion to AD, thereby suggesting its potential use as biomarker for AD progression.

## Introduction

Alzheimer’s disease (AD) is a progressive neurodegenerative proteinopathy characterized by deposition of amyloid β (Aβ) and hyperphosphorylated tau protein in the brain of patients. The pathology observed in AD begins years, or even decades, before the appearance of clinical symptoms. Thus, identification of biomarkers reporting on pathways modulating AD pathology in asymptomatic individuals at-risk is of paramount importance to define target groups for early prevention strategies once these become available. This, however, has been proven to be a major challenge as several, partially unknown, pathways contribute to the pathology leading to neurodegeneration, cognitive decline and finally dementia (De Strooper and Karran, 2016). Unfortunately, current validated biomarkers inform on the neuropathological hallmarks of the disease following the amyloid cascade hypothesis leaving other pathways uncovered (Jack et al., 2018). This assumption receives further support from disappointing results from amyloid-specific therapies in AD.

Dementia stage in AD is the culmination of a series of events that begin with a complex interplay between genetic and environmental susceptibility factors years before cognitive symptoms become apparent. This interplay triggers a sequence of pathological changes which involves process altering Aβ homeostasis, as well as processes beyond amyloid such as vascular changes, neuroinflammation and age-related factors relevant for reserve and resilience of the brain (De Strooper and Karran, 2016). Given the difficulty linked to the search for biomarkers informing on these pathways in humans, research has turned into model organisms to identify and to characterize conserved pathogenic pathways and molecules that could serve as biomarkers for AD (Wang et al., 2021). Herein, a promising animal model is the McGill-R-Thy1-APP rat (Leon et al., 2010) expressing the human amyloid precursor protein (APP) with the Swedish and Indiana mutations responsible for familial AD in humans. The hemozygous Tg ± rats do not develop extracellular plaques, but show intraneuronal accumulation of Aβ in cortex and hippocampus (Leon et al., 2010; Iulita et al., 2014), a similar feature was described in the human brain at early stages of AD amyloid pathology (Christensen et al., 2010). Moreover, these animals show accumulation of SDS-resistant Aβ oligomers (∼30 kDa) from 6 months onwards (Galeano et al., 2014); synaptosomal bioenergetic defects (Martino Adami et al., 2017a) and cognitive impairments in different hippocampal-dependent behavioral tasks (Leon et al., 2010; Galeano et al., 2014; Iulita et al., 2014; Martino Adami et al., 2017a; Martino Adami et al., 2017b; Habif et al., 2021) resulting and interesting model of early AD-amyloid pathology. The homozygous Tg+/+ rats show the full AD-like-amyloid pathology, accompanied by neuroinflammation and cognitive impairment, reflecting stages of late AD (Leon et al., 2010). While the Tg rat model has been extensively used to explore stages of AD pathology and validation of experimental therapeutic candidates, studies linking the metabolic profiles in hippocampus in association with the degree of amyloid pathology are still lacking. Furthermore, translational research is still needed to define whether findings made in the McGill-R-Thy1-APP rat can also be seen in AD patients.

Identification of novel biomarkers covering pathogenic pathways beyond classic amyloid cascade pathways will derive in better clinical diagnosis, particularly at preclinical stage of the disease. Recent developments in sensitivity and specificity of proteomics and metabolomics technologies have made it possible to identify different molecules targeting these additional pathological pathways. Thus, for example, cerebrospinal fluid (CSF) and blood levels of the neurofilament light chain (NfL) have been used as a sensitive biomarker for neuroaxonal damage that can monitor neurodegeneration and progression of Alzheimer’s disease dementia, albeit not specific (Norgren et al., 2003; Gaiottino et al., 2013). While most reports have been done using data derived from mouse models for neurodegenerative diseases (Wilkins and Trushina, 2017), few reports have been focused on the McGill-R-Thy1-APP rat model (Nilsen et al., 2012; Nilsen et al., 2014b).

Consequently, this study aimed to characterize metabolic abnormalities in the hippocampus of homo- and hemizygous McGill-R-Thy1-APP rats by using Nuclear Magnetic Resonance (^1^H-NMR) spectroscopy. Promising findings in the rat were followed up in human plasma samples by Gas Chromatography Electron Impact Mass Spectrometry (GC-EI-MS) to explore their potential utility as AD biomarkers.

## Materials and methods

### Rat model

Transgenic (Tg) McGill-R-Thy1-APP rats (Leon et al., 2010) were provided to Fundación Instituto Leloir (FIL) by The Royal Institution for the Advancement of Learning/McGill University, Montreal, Canada, and an in-house colony was established at FIL. Rats’ genotypes were determined by real time qPCR as previously described (Galeano et al., 2014). To avoid the litter effect, groups were made up of pups from three to four different litters. Homozygous (Tg+/+), hemizygous (Tg+/-), and littermates’ wild type (WT) control animals were maintained in polycarbonate cages in a temperature-controlled animal facility with a 12-h dark/light cycle and allowed to consume standard diet and water *ad libitum*. Only 9-month-old male rats were used for experiments to avoid any potential effects of female estrus cycle. All experimental procedures were performed in accordance with the guidelines of ARRIVE and OLAW–NIH. The protocol was approved by the local animal care committee (CICUAL # A5168-01).

### Rat hippocampal tissue collection

Rats were anesthetized with ketamine (50 mg/kg) and xylacine (10 mg/kg), placed under a guillotine blade, decapitated and brains quickly removed. Sacrifices were carried out during the morning. Hippocampi were dissected and processed as described in the Supplementary Figure S1 minimizing the time between sacrifice and tissue freeze.

### Human plasma samples

Samples were selected from the German study on Aging, Cognition and Dementia (AgeCoDe) biobank (Ramirez et al., 2015). The original study protocol was approved by the local ethics committees at the following German institutions: University of Bonn; University of Hamburg; University of Duesseldorf; University of Heidelberg/Mannheim; University of Leipzig and the Technical University of Munich. Written informed consent was obtained from all participants. The main assessment instrument at all visits included the Structured Interview for Diagnosis of Dementia of Alzheimer type, Multi-infarct Dementia and Dementia of other etiology according to DSM-IV and ICD-10 (SIDAM), and diagnosis of AD was established according to the NINCDS-ADRDA criteria for probable AD (McKhann et al., 1984; Zaudig et al., 1991).

This is a longitudinal study, where participants were recruited in primary care centers in six German cities. Inclusion criteria were to be at least 75 years old and cognitively healthy according to the general practitioner’s judgment. Every ∼18 months interval participants are followed-up with personal interviews and neuropsychological assessments. To date, nine follow-ups (FUs) were completed, but results from the last one are still in process. Blood samples were obtained at the third visit, processed and store at -80°C. For this study the third visit is considered the baseline. Controls (*n* = 189) remained cognitively unimpaired until the last FU, and were 83.6 ± 3.1 years old, 64.0% female and 20.6% Apolipoprotein E4 (*APOE4*) carriers. In this report, participants who converted to AD at baseline were denominated *incident* AD (*n* = 68), and participants with diagnosis of AD before the baseline, were denominated *prevalent* AD (*n* = 29). Participants with *incident* AD were 86.0 ± 3.6 years old, 64.7% female and 33.8% *APOE4* carriers; and those with *prevalent* AD, were 84.2 ± 3.1 years old, 75.8% female and 37.9% *APOE4* carriers. Subjects converting to AD in the next three visits following baseline (FU1, FU2 and FU3) were included in the analysis. At FU1 there were 25 participants with mean age of 84.8 ± 3.5 years old, 80% women, and 28% *APOE4* carriers; at FU2 there were 37 participants with mean age of 83.6 ± 2.6 years old, 67.6% women, and 32.4% *APOE4* carriers; and at FU3 there were 23 participants with mean age of 82.7 ± 2.6 years old, 60.9% women, and 21.7% *APOE4* carriers.

### Expression of aβ isoforms in rat hippocampus

To quantify human Aβ 38/40/42 MSD^®^ V-PLEX PLUS Aβ Peptide Panel one kit was used following the manufacturer’s instructions. Methodology is described in the SI.

### Untargeted nuclear magnetic resonance (NMR) spectroscopy

Frozen rat hemi-hippocampus were homogenized with a teflon-glass grinder in 2 ml ice-cold 80% methanol (Nagana Gowda et al., 2018) and centrifuged at 4°C for 10 min at 15000 xg. Supernatants were collected, dried in a Savant SpeedVac (Thermo Scientific) and solubilized in .5 ml sodium phosphate buffer (100 mM dissolved in D_**2**_O, pH = 7.4), supplemented with 3-trimethylsilyl-[2,2,3,3,-2H4]-propionate (TSP, final concentration .33 mM) as chemical shift reference. Sample sizes for NMR experiments were chosen using an analysis based approach, MetSizeR (Nyamundanda et al., 2013). All NMR experiments were performed at 298 K on a Bruker Avance III spectrometer operating at a proton frequency of 600.3 MHz. ^1^H-NMR 1D spectra were acquired using a standard Bruker 1D NOESY pulse program with pre-saturation during relaxation delay and mixing time, and spoil gradients (noesygppr1d). The following experimental parameters were used in all measurements: 256 scans, 1.85 s relaxation delay, 1.36 s acquisition time, 20 ppm spectral width, 10 m mixing time, and 32 K acquisition points. The NMR data were zero-filled, Fourier transformed, phase corrected using NMRPipe and converted to a Matlab-compatible format for further processing and analysis. All spectra were referenced to TSP (1H *δ* = 0 ppm) and submitted to water peak elimination, baseline correction, normalization, and scaling. The assignment was achieved using the freely available electronic databases HMDB and BMRB, and subsequently confirmed by 2D spectra including heteronuclear single quantum coherence (HSQC) and total correlation spectroscopy (TOCSY) (Supplementary Table S1). 2D ^1^H–^1^H TOCSY spectra were collected with N1 = 512 and N2 = 2048 complex data points. The spectral widths for the indirect and the direct dimensions were 9,615.4 and 9,604.9 Hz, respectively. The number of scans per t1 increment was set to 36. The transmitter frequency offset was 4.7 ppm in both ^1^H dimensions. 2D ^13^C-^1^H HSQC spectra were collected with N1 = 512 and N2 = 2048 complex data points. The spectral widths for the indirect and direct dimensions were 24,906.9 and 12,019.2 Hz, respectively. The number of scans per t1 increment was set to 256. The transmitter frequency offset was 70 ppm in the ^13^C dimension and 4.7 ppm in the ^1^H dimension. The estimated detection limit for ^1^H NMR (at 600 MHz) is dependent on the compound and varies between 1–10 µM.

### Measurement of NAD+ and NADH in rat hippocampal tissues

NAD+/NADH levels were measured using NAD+/NADH assay kit from Abcam (ab65348) as described in SI.

### Determination of enzymes transcript levels of NAD rate-limiting and NAD salvage pathway

MRNA levels of NAMPT (rate-limiting); NMNAT (NAD-generation) and CD38, PARP1, PARP2 and Sirt 3 (NAD-consuming) enzymes were assessed by qRT-PCR as described in the SI.

### Targeted Gas Chromatography Electron Impact Mass Spectrometry (GC-EI-MS)

 Human plasma samples were thawed on ice, and 100 ul were extracted with 900 ul of cold extraction buffer containing 40:40:20 methanol:acetonitrile:water [v:v:v]. After 30 min in an orbital mixer at 4°C, samples were sonicated for 10 min in an ice-cooled bath-type sonicator and centrifuged for 10 min at 16000xg at 4°C. Supernatants were collected and dried in a SpeedVac until complete dryness. Standard curves of Nam were prepared with concentrations ranging from .005 to 50 ug/ml (expected limit of detection .1–.5 ng/ml). Standards were processed in the same way as samples. Dried down samples and standards were derivatized using methoxyamine and MSTFA/FAMEs solution (N-methyl-N-trimethylsilyl-trifluoracetamid/Fatty acid methyl esters) following standard procedures (Lisec et al., 2006; Caldana et al., 2013). After that, samples were analyzed in a GC-EI-MS (Q Exactive GC Orbitrap system, ThermoFisher) using a 30-m DB-35M capillary column. Representative fragments from the GC-EI-MS analysis of Nam were extracted using TraceFinder (Version 4.1, ThermoFischer) and quantified using the linear range of the obtained standard curve. All analysis were performed using peak areas, transformed into Z-scores, for easier comparison among experiments.

### Statistical analysis

The normalized NMR spectral areas (AUC) of assigned metabolites were subjected to Pareto scaling and analyzed by multivariate analysis using MetaboAnalyst 5.0 (Chong et al., 2018) (Supplementary Figure S1). The statistical significance was assessed by one-way ANOVA, Fisher’s LSD were performed for all post-hoc tests taking *p* < 0.05 as significant.

Data of NADH and NAD + levels were analyzed by one-way ANOVA tests followed by post-hoc Tukey’s multiple comparisons tests.

Statistical analysis and plots of data from GC-EI-MS experiments performed with human plasma were done using R-project v. 4.0.0 (https://www.R-project.org) and R-studio v.1.2.5042 (http://www.rstudio.com/). Normal distribution was visualized using qqnorm plots, and outliers (defined as mean ± 3 standard deviation) were eliminated from the analysis (*n* = 2). For easier comparison among experiments in human samples a Z-score standardization was applied and subsequently data were analyzed by one-way ANOVA tests followed by post-hoc Tukey’s multiple comparisons tests. In all cases, assumption of normality was examined using Kolmogorov-Smirnov or Shapiro–Wilk tests. A probability equal or less that 5% was considered as significant. All analyses were carried out using GraphPad Prism for Windows (version 7.0).

Linear regression models adjusted for sex, age and apoe4 were used to estimate the association of Nam levels in cases vs. controls in the *discovery* and *replication* experiments, as well as in FUs groups. Meta-analysis was performed using the R-package “metafor” (Viechtbauer, 2010) and visualized with the general function forest. The cox proportional hazards regression model, which relates time dependent variables, time dependent strata, and multiple events per subject, were performed with the R-package “survival” (Therneau, 2020) and “survminer” (Kassambara, 2021). Samples of paritcipants converting toAD at FU1-3 were included, time variable was time to conversion to AD in years, and the event per subject was conversion (no = 0, yes = 1). Proportional hazard assumption was tested by Schoenfeld’s test, and consequently two cox regressions were performed with a split-time = 2.5 years.

## Results

### Comparison of ^1^H-NMR metabolomics profiles of Tg and control rats

**FIGURE 1:**
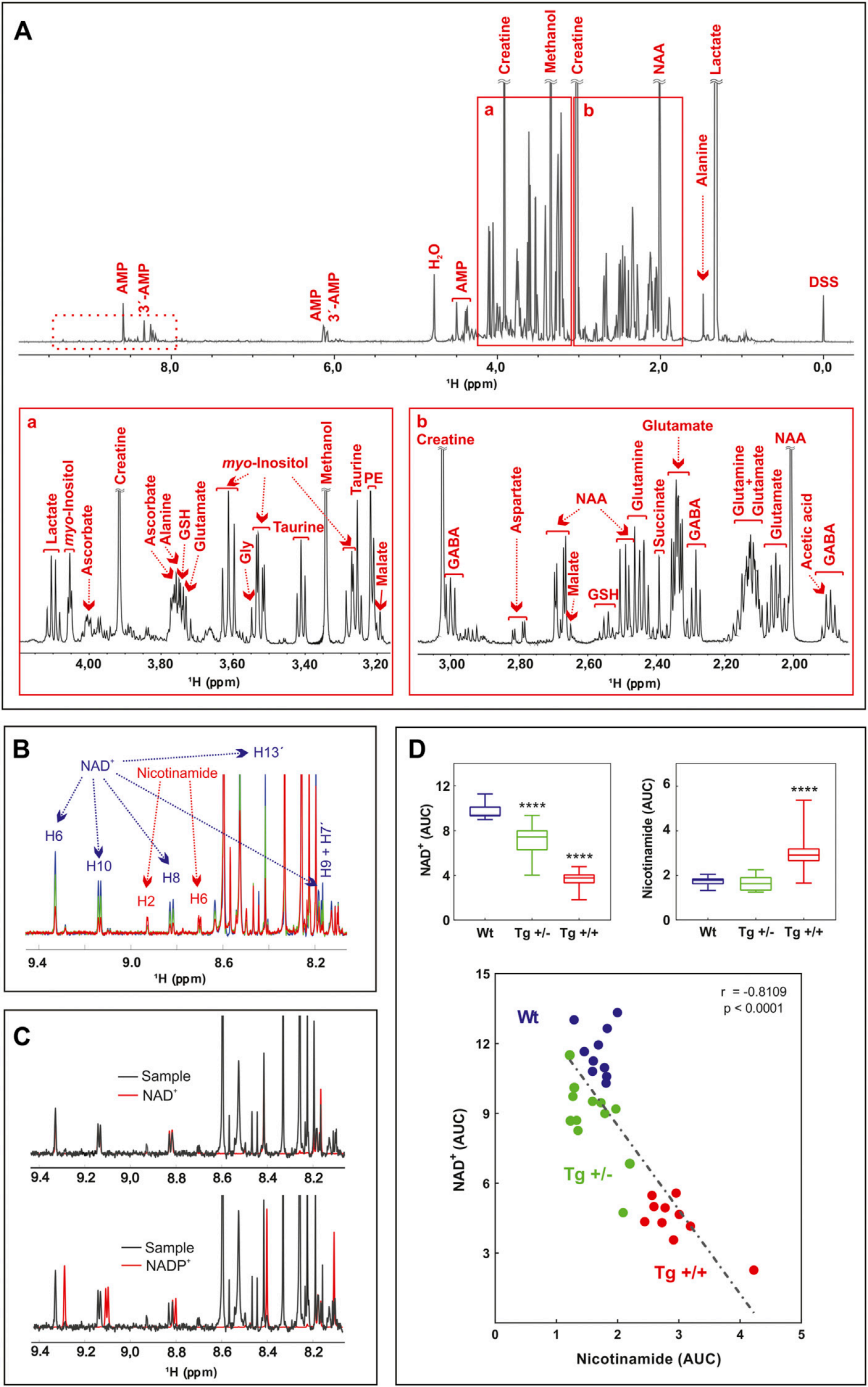
Untargeted ^1^H-NMR metabolomics of hippocampus of AD-like amyloid pathology transgenic rats **(A)** Typical 600 MHz ^1^H-NMR spectrum of WT rats, representative of all the registered spectra. Assigned resonances of specific metabolites are indicated in red. Expanded views of the spectrum between 3.1–4.4 ppm **(A)** and 1.7–3.2 ppm **(B)** are shown **(B)** Overlaid of averaged ^1^H-NMR spectra of WT rats (blue) (*n* = 10), Tg +/- (green) (*n* = 12) and Tg+/+ (red) (*n* = 10) in the 9.5–8.0 ppm zone (dashed box in A). The resonances assigned to NAD and Nam protons are indicated **(C)** Overlaid of representative ^1^H-NMR spectra of WT rats (black, sample), NAD standard (red, upper panel) and NADP standard (red, lower panel) **(D)** Lower panel: correlation between NAD and Nam levels of samples analyzed. The AUC of H6 of NAD and H2 of Nam were plotted (*n* = 32). The linear regression (dashed line), the Pearson’s correlation coefficient and the *p*-value (two tailed) are shown. Upper panels: differences in the AUC of NAD (left) and Nam (right) among groups (WT, blue; Tg+/-, green and Tg+/+, red) were analyzed by one-way ANOVA. Significant differences are indicated accordingly to Fishers´s LSD test. ****p < 0.0001.

In this report we did not assess the cognitive status of Tg rats. However, it was previously reported by us and others that from 3 to 9 months of age, Tg rats show impairments in learning and spatial reference memory (Galeano et al., 2014; Wilson et al., 2017a), in long-term memory of inhibitory avoidance to a foot-shock, in novel object recognition memory and social approaching behavior (Habif et al., 2021), in cued fear-conditioning recall (Wilson et al., 2017a), and associative learning (Wilson et al., 2017b). Studies by Leon et al. (2010) and Iulita et al. (2014) established that 13-month-old Tg+/+ rats show marked cognitive impairments, while Tg ± rats perform intermediately between homozygous and WT genotypes. To determine Aβ-associated shifts in brain metabolites, we first performed a highly sensitive multiplex ELISA to quantify total Aβ levels within the hippocampus of a sub-set of Tg rats (*n* = 3–7). The median value of the concentration of Aβ40 showed nearly 4-fold increase in Tg+/+ [54.4 [pg/mg] (IQR: 31.8–55.7)] vs. Tg+/− [13.8 [pg/mg] (IQR: 9.8–31.1), *p* = .048]. For Aβ42, the increased was more than 20-fold [48.8 [pg/mg] (IQR: 16.7–153.4)] vs. Tg+/− [2.4 [pg/mg] (IQR: 2.0–7.5), *p* < 0.035]. These results confirmed the impact of the two copies of human mutant APP transgene on the accumulation of cerebral amyloid. To identify metabolic changes in pathways relevant for the hippocampus of the McGill-R-Thy1-APP rat, we carried out untargeted ^1^H-NMR metabolomics on methanol-extracted samples from freshly isolated tissues. A total of 26 compounds were detected and identified (residual methanol was excluded from the analysis), including mainly amino acids, carboxylic acids, and nucleotides (Figure 1A; Supplementary Table S1).

Principal component analysis (PCA) and partial least square-discriminant analysis (PLS-DA) were performed in order to detect the differences among control and Tg rats (Supplementary Figure S1). Although the 95% confidence intervals generated for each group overlapped in the PCA score plots, a pronounced separation was observed for Tg+/+. Supervised PLS-DA was in line with PCA and gave a clearer separation between groups (Supplementary Figure S1). Nine metabolites were found to be significantly altered in Tg rats vs. control (Table 1).

**TABLE 1:** Hippocampal metabolites detected by ^1^H-NMR spectroscopy that showed significant differences between control and Tg rats.

Metabolite	FRD			
	Tg+/-	Tg+/+	Tg−/+	Tg+/+
NAD	2,50E-06	8,36E-11	↓	↓
Nam	>0.05	3,20E-05	=	↑
Taurine	2,39E-06	2,70E-05	↓	↓
Valine	1,71E-02	>0.05	↓	=
GSH	>0.05	2,08E-02	=	↑
Tyrosine	>0.05	2,47E-02	=	↑
NAA	3,55E-02	>0.05	↓	=
Creatine	3,55E-02	>0.05	↓	=
Glutamate	4,59E-02	4,80E-02	↓	↓

Differences in the AUC, of metabolites were analyzed by one-way ANOVA, with Fisher’s LSD, post-hoc test; FDR, false discovery rate. Arrows indicate significant increase (up) or decrease (down) with respect to control rats. = , no change from control rats. NAD, nicotinamide adenine dinucleotide; Nam, nicotinamide; GSH, glutathione; NAA, N-acetylaspartate.

**FIGURE 2:**
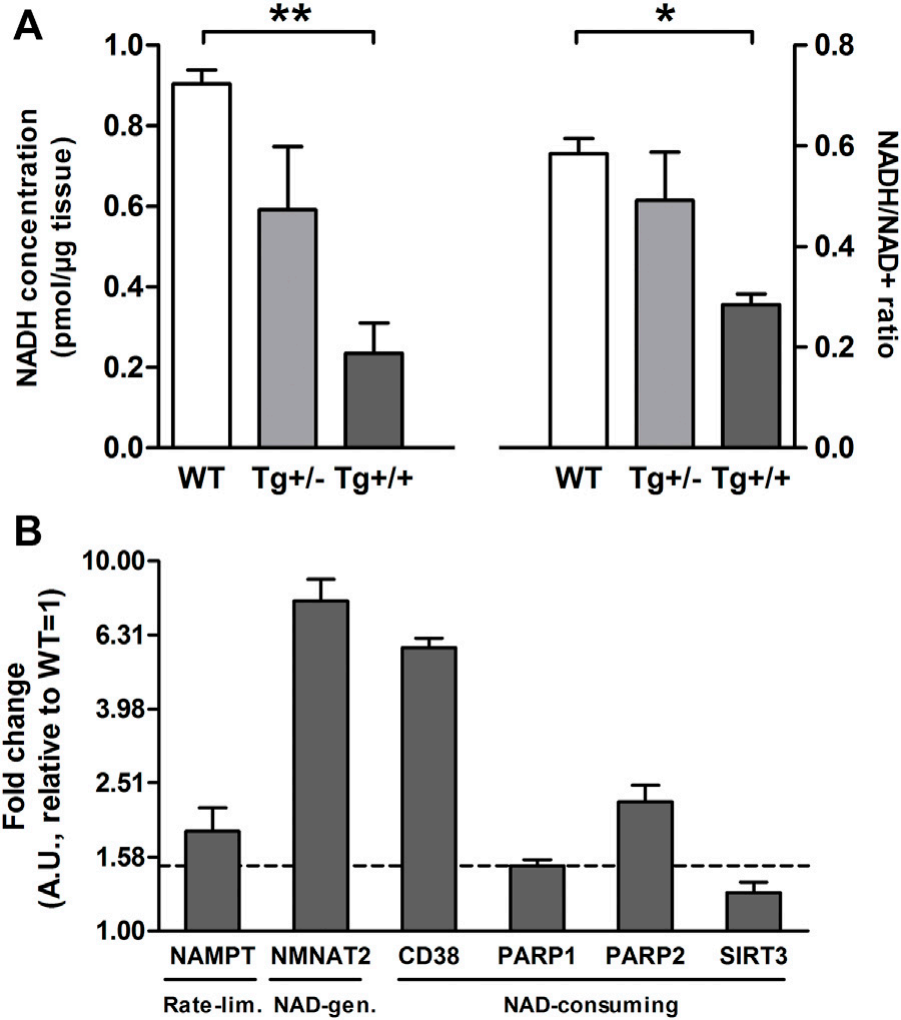
Hippocampal Aβ deposition alters the brain NAD + metabolism **(A)** Bars show mean ± SEM levels of NADH (left panel) and NADH/NAD + ratio (right panel) in hippocampal homogenates of control (WT; *n* = 3), hemizygous (Tg+/; *n* = 3) and homozygous (Tg+/+; *n* = 3) transgenic rats. One-way ANOVA tests and *post hoc* analyses revealed that Tg+/+ showed significantly lower levels of NADH (F_(2, 6_) = 10.76, *p* = 0.01; post-hoc: **p < 0.01) and NADH/NAD + ratio (F_(2, 6_) = 6.71, *p* = 0.02; post-hoc:*p < 0.05) compared with those observed in WT **(B)** Transcript levels of rate-limiting (NAMPT), NAD + -generation (NMNAT2) and NAD + consuming enzymes (CD38, PARP1, PARP2 and SIRT3) in hippocampal homogenates of Tg+/+ rats. Each bar represents the mean ± SEM of at least three independent experiments performed by triplicate for each sample normalized by GAPDH or Eukaryotic Translation Elongation Factor 1 Alpha 1 (EEF1A1). The mean ± SEM relative to WT (=1) is shown. Values above the dashed line (+1.5) were considered different from WT (=1).

Interestingly, most of them (i.e., taurine, glutathione, tyrosine, and glutamate) have been previously reported in several studies on metabolomics performed in biological samples from the CNS, both in animal models and in AD patients (Altine-Samey et al., 2021). However, the PLS-DA loadings with the larger absolute weights and the highest VIP scores were NAD and Nam (Supplementary Figure S1). These two metabolites showed significant differences between genotypes (Figures 1B, D). Standard runs analysis was performed in order to confirm de identity of NAD in contrast to its related metabolite Nicotinamide Adenine Dinucleotide Phosphate (NADP) (Figure 1C). As previously described for these key molecules in the NAD salvage-pathway, Nam and NAD levels showed an inverse relationship (Figure 1D; Supplementary Figure S1). Since NMR analysis cannot differentiate between NADH and NAD+, and taking into account that NADH levels decrease as a function of age and that the ability to regenerate NADH drops sharply in aged brain (Lautrup et al., 2019) we quantified NADH and NAD + by a colorimetric kit and found that homozygous rats (Tg+/+) showed significantly lower levels of NAD+ and NADH compared with those observed in WT. In contrast, hemizygous rats (Tg+/-) showed an intermediate level that did not reach significance neither with WT nor with Tg+/+ (Figure 2A). In this regard, NADH/NAD + ratio was significantly lower in the Tg+/+ as compared to WT and Tg +/- (Figure 2B) suggesting a clear alteration of the redox state in the brains of Tg+/+ rats, which is probably still incipient in the Tg ± ones. To explain alterations in Nam and NAD+/H levels observed in Tg+/+ brains we first evaluated transcript levels of Nicotinamide phosphoribosyltransferase (NAMPT) the rate-limiting component in the NAD + rescue pathway (Garten et al., 2015) and found a slight increase (1.85 ± 0.29) as compared with the control group (WT = 1) (Figure 2B). In addition, gene expression of NAD + -generating enzyme nicotinamide mononucleotide adenylyltransferase (NMNAT2) and NAD + -consuming (CD38, PARP1, PARP2 and SIRT3) enzymes (Okabe et al., 2019) were also assessed. We detected increments greater than 1.5 fold-change in NMNAT2 (7.8 ± 1.12), CD38 (member of the cyclic ADP-ribose synthase family) (5.83 ± 0.35) and PARP2 (member of the ADP-ribose transferases family) (2.23 ± 0.24). Whereas transcript levels of PARP1 and SIRT3 (sirtuin) were unaffected (1.5 ± 0.06 and 1.27 ± 0.08, respectively) (Figure 2B). Based on these results, expression of rate-limiting enzyme in Tg+/+ seems to be slight different from WT, while NAD + -consuming and the NAD + -generating pathways seem to be activated in Tg+/+ brain suggesting potential disturbance of the NAD + rescue pathway following the ongoing amyloid pathology. While central disturbance in NAD + metabolism in Tg rats was observed, its translation to peripheral tissue was unclear.

### Plasma nam levels as a potential biomarker of AD

**FIGURE 3:**
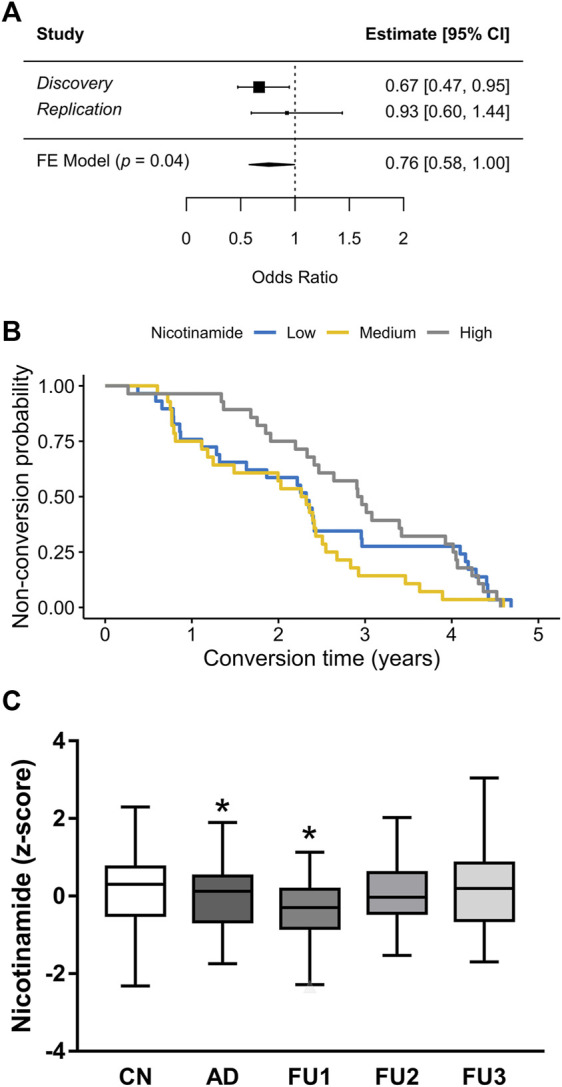
Plasma levels of Nam in association with AD **(A)** Meta-analysis forest plot of Nicotinamide plasma levels in human samples. Discovery experiment includes 68 cases and 93 controls. Replication experiment includes 29 cases and 93 controls. Estimates are in Odds Ratios; CI, confidence interval; FE Model, fixed effects meta-analysis results. **(B)** Kaplan-Meier conversion to AD survival of 85 participants after blood test for Nicotinamide, stratified in high, medium or low levels. High levels of Nicotinamide seem to be a predictor of dementia survival for 2.5 years (HR = 0.73, *p* = 0.04) **(C)** Box plots represent the normalized GC-EI-MS spectral areas of Nicotinamide in human plasma of CN (cognitive normal) subjects (*n* = 189); AD (*n* = 85) patients and FU1 (*n* = 25); FU2 (*n* = 37) and FU3 (*n* = 23) participants.*p < 0.05.

The results in the brain of the rat prompted us to explore whether these findings can be translated to humans. Herein, we focused our analysis on plasma because it might offer a promising alternative for biomarker in blood. Since measure of NAD in clinical practice is methodologically complicated because of its size (665 Da) and its stability in chromatography solvents, Nam levels were measured. To this aim, targeted detection of Nam was performed in human plasma samples from the longitudinal study AgeCoDe using GC-EI-MS. First, we compared whether Nam plasma levels of 68 participants with AD dementia (AD) showed statistical differences compared to 93 cognitively normal (CN) participants and found that Nam levels were significantly reduced in cases compared to CN (odd ratio (OR) = 0.67, *p* = 0.02, Figure 3A). In an independent replication sample drawn from AgeCoDe, including 96 CN and 29 AD, Nam showed the same trend (OR = 0.93) which, however, did not reach significance (*p* = 0.7, Figure 3A) probably due to the small number of cases analyzed. The meta-analysis of both samples confirmed the protective effect of plasma levels of Nam (OR = 0.76, *p* = 0.04, Figure 3A).

### Nam plasma levels as a prospective biomarker of AD conversion

To better analyze our results we explored whether Nam plasma levels, measured at baseline, were associated with the time to conversion to AD. Consequently, participants were included in the analysis if they have available data on plasma levels of Nam and converted to AD at any of the next three follow-ups (FU) for which data was available [(FU1) = 0.94 ± 0.35 years after baseline; FU2 = 2.43 ± 0.38 years after baseline; FU3 = 4.13 ± 0.37 years after baseline]. For the analysis, the impact of Nam levels was stratified in tertiles (high, medium and low) and their effect on time to conversion was visualized by *Kaplan-Meier* survival curves (Figure 3B) and analyzed by Cox regression models. This analysis showed that only the higher levels of Nam are associated with a later conversion to dementia (hazard ratio (HR) 0.73, *p =* 0.04). However, we also observed that the HR is not proportional over time (curves intersect). Thus, while a person with Nam levels in plasma within the high tertile has 27% risk reduction of progressing to AD within the next 2.5 years, this HR is lost afterwards. Supporting this finding, we observed that only participants progressing to AD at FU1 showed significantly lower levels of Nam compared with CN (*p =* 0 .04, Figure 3C).

## Discussion

Alzheimer’s disease is a complex phenotype involving several pathogenic pathways leading to a metabolic imbalance already at early stages of the disease before symptom become apparent. Relevant pathways altered in AD include lipid and amino acid metabolism, as well as dysregulation of the glucose metabolism and mitochondrial dysfunction guiding to energetic imbalance and oxidative stress (Yan et al., 2020). Using untargeted ^1^H-NMR spectroscopy, we observed in hippocampus of the McGill-R-Thy1-APP rat a significant reduction of NAD level in Tg rats compared to their healthy littermates at 9 months of age suggesting an energetic imbalance in the Tg rats.

NAD + and related metabolites are critical compounds essential to adaptive stress responses and cell survival. It was well established that PARP-1 (a NAD + consuming enzyme) functions as a DNA repair enzyme under intense DNA damage as is the case of late AD brain neuropatholgy. It was postulated that PARP-1 activity depletes neurons of NAD+ and ATP leading to neuronal death by a caspase-independent mechanism that shares characteristics of apoptosis and necrosis (known as Parthanatos), recently reviewed by Salech et al. (2020). Interestingly, Nam is a well-known inhibitor of PARP-1. It is of note that McGill-R-Thy1-APP rats used in this study lack neuropathology of late-AD brains and PARP-1 transcript levels in Tg rat brains are similar to control animals, suggesting that Parthanatos is not operative in this animal model of brain amyloidosis. Considering the number of enzymes and transcription factors sensitive to the redox potential, NAD+/H redox state acquires pathophysiological relevance for aging and neurodegenerative diseases (Verdin, 2015; Fang et al., 2017). While several studies in mouse models for AD have shown the relevance of the NAD(P)+/NAD(P)H homeostasis in the brain, especially in hippocampus and cortex (Ghosh et al., 2012; Dong and Brewer, 2019; Dong et al., 2019), few reports have been published on the role of the NAD(P)+/NAD(P)H homeostasis in the McGill-R-Thy1-APP rats. A previous *in vivo* study using Magnetic Resonance Spectroscopy (^1^H-MRS) identified in tissue derived from hippocampus and frontal cortex of McGill-R-Thy1-APP rat significant difference in levels of several metabolites compared to the WT littermates (Nilsen et al., 2012). Herein, the Tg+/+ rats, compared to WT rats, showed lower levels of glutamate, GABA, N-acetylaspartate (NAA) and elevated myo-inositol and taurine. These differences become apparent during the progression of amyloid pathology in Tg+/+ in time window of 6 months between three-to 9-months of age. Previously, the NAA and myo-inositol findings were reproduced in dorsal hippocampus tissue derived from this rat model, though only in males Tg+/+ rats (Nilsen et al., 2014b). Metabolites identified in these studies suggested brain damage and mitochondrial dysfunction that might be gender specific. In line with this report, we were able to replicate in part these previous differences using brain tissue from this rat model. Minor differences might derive from dissimilarities in the techniques used in previous studies compared to ours (*in vivo* ^1^H-MRS vs *ex vivo* ^1^H-NMR). By using *in vivo* ^1^H-MRS the regional concentration of low molecular weight metabolites can be measured non-invasively. Conversely, *ex vivo* ^1^H-NMR spectroscopy detects only hydrophilic metabolites extracted from tissue homogenates. Hence, both approaches might be complementary for the identification of neurochemical processes related to AD pathology and its progression over time. Our findings on NAD receive further supports from ^1^H- and ^13^C NMR spectroscopy and HP-LC experiments done in cingulate cortex derived from aged McGill-R-Thy1-APP (15-month-old) that showed decreased levels of NAD + in Tg rats compared to WT (Nilsen et al., 2014a).

In correlation with our findings, experimental evidence supports a protective effect of NAD + supplementation on cognitive deficits in AD models (Gong et al., 2013; Liu et al., 2013). It was previously reported (Xing et al., 2019) in hippocampal tissue of 6-month-old APPswe/PS1Δe9 transgenic mice decrements of NAD-generating enzyme (NAMPT) levels which were reverted by the administration of NAD, suggesting that increasing NAMPT expression levels may promote NAD production. Our results showed a slight increase of NAMPT transcript levels in Tg+/+ as compared to control rats (1,8 fold-change as compared to WT = 1). It is of note that NAMPT expression is induced by inflammatory signals and is considered a biomarker of chronic and acute inflammatory disease (Audrito et al., 2020). In this regard, hippocampal accumulation of Aβ in Tg+/+ rats may act as an alarmin triggering proinflammatroy cytokines (Wilson et al., 2018) and promoting increments of NAMPT transcript levels as reported here. Consequently, we postulate the possibility to use NAD + metabolites as peripheral biomarkers for AD. In line with this hypothesis, our study identified a significant lower level of plasma Nam in AD patients compared to healthy controls. This difference was also seen before the patients progressed to AD. Thus, Nam levels in plasma could serve as biomarker for progression to AD. However, risk reduction associated to high levels of Nam is lost after 2.5 years, meaning it is only valid in the close proximity to its assessment. The relevance of our observation is reinforced by a recent report showing that by untargeted metabolomics 308 CSF metabolites from 338 individuals were identified and associated using principal components (PCs) analysis with CSF total tau (t-tau), phosphorylated tau (p-tau), Aβ42, and Aβ42/40 ratio. Employing linear regression models 5 PCs were significantly associated with CSF p-tau and t-tau and 3 PCs with CSF Aβ42. Pathway analysis suggested that these PCS were enriched in six pathways, including metabolism of caffeine, nicotinate and Nam. (Dong et al., 2022).

In addition to the role of Nam as a potential biomarker for AD progression Nam may be also involved in AD onset. In this regard, a new mechanism of AD induction was recently postulated in which NAD depletion due to inadequate levels of Nam may have a relevant role in neuronal damage. On this point, the dietary habits in the aging characterized by low fruits and vegetable consumption and the presence of visceral fat which secretes visfatin, an inflammatory adipokine that deplets blood Nam, may explain why many people do develop AD due to lifestyle (Adams, 2021). However, research in humans has shown that plasma levels of NAD + decrease while levels of Nam increase significantly with age (Clement et al., 2019). These data have fueled several clinical trials of NAD + precursors, which still, produced inconsistent results (Rainer et al., 2000; Demarin et al., 2004).

The utility of Nam as a treatment for prevention of AD is still on debate. It was reported in animal models of AD that dietary supplements of Nam can increase the amount of (NAD) (+) in the brain, reduce the production of Aβ, and slow the decline of cognitive function. While Nam has shown promise in the treatment of AD, a Phase II Clinical Trial failed to demonstrate that Nam improves cognitive function in subjects with mild to moderate AD over 24 weeks. The lack of efficacy of Nam was explained due to several factors including a low sample size (*n* = 15); inclusion of subjects with moderate AD, and a relatively short treatment phase (Phelan et al., 2017). Currently few more human clinical trials are ongoing to evaluate the safety concerns of Nam supplementation however the outcomes are yet to be available (Nadeeshani et al., 2021).

We are aware that this study has also limitations. We only employed male Tg rats due to the well known effect that estrous cycle has on biochemical parameters, increasing intra-group variability. This is a clear impediment for generalizing results. However, in the experiments with human samples both genders were included. Finally, the replication analysis on human plasma did not reach significance. Since the effect goes in the same direction and the meta-analysis is still significant, this could be a minor problem. A replication in independent cohorts is necessary to validate the potential use of plasma levels of Nam as biomarker for AD progression.

## Conclusion

In summary, our study provides additional supporting evidence indicating that hippocampal Aβ burden and/or hAPP processing is associated with the degree of NADH/NAD + shift in McGill-R-Thy1-APP rat brain. Although this information cannot infer causal direction it offers a different perspective on the Aβ-mediating mechanisms involved in brain energy dysfunction observed in AD. Besides, our findings indicate that plasma Nam content has a potential role as short-term AD risk biomarker. Nevertheless, further studies in larger cohorts and independent populations of patients will be needed to confirm our results and the potential use of Nam as peripheral biomarker. To our knowledge, this is the first report showing a significant decrease of Nam plasma levels in people with AD that is observed couple of years before conversion, thereby suggesting its potential use as biomarker for progression.

## Data Availability

The raw data supporting the conclusions of this article will be made available by the authors, without undue reservation.
