# Supplementary material for: Nicotinamide as potential biomarker for Alzheimer’s disease: A translational study based on metabolomics
Source: Front Mol Biosci. 2023 Jan 6;9:1067296. doi: 10.3389/fmolb.2022.1067296 (PMC9853457; doi:10.3389/fmolb.2022.1067296)
Supplement: Supplementary file 1 [file DataSheet1.DOCX]

**Supplementary Information**

**Nicotinamide as potential biomarker for Alzheimer’s disease: a translational study based on metabolomics**

María C. Dalmasso, PhD; Martín Arán, PhD; Pablo Galeano, PhD; Silvina Perin MS; Patrick Giavalisco PhD; Pamela V. Martino Adami, PhD; Gisela V. Novack MS; Eduardo M. Castaño, MD; A. Claudio Cuello, DSc; Martin Scherer MD; Wolfgang Maier MD; Michael Wagner PhD; Steffi Riedel-Heller MD; Alfredo Ramirez, PhD; Laura Morelli, PhD

**Supplementary Methods**

**Rat hippocampal tissue collection**. Rats were anesthetized with ketamine (50mg/kg) and xylacine (10mg/kg), placed under a guillotine blade, decapitated and brains quickly removed. Hippocampi were dissected on an ice-cold plate, and divided into left and right hemispheres. Each hippocampus was immediately and independently frozen in liquid nitrogen, and stored at -80 °C until used.

**Expression of Aβ isoforms in rat hippocampus.** To quantify human Aβ 38/40/42 MSD® V-PLEX PLUS Aβ Peptide Panel 1 kit was used following the manufacturer’s instructions. Briefly, hippocampus of Tg rats were processed with illustra triplePrep kit (GE) and Individual protein samples were loaded onto MULTI-SPOT® microplates pre-coated with antibodies specific to the C-termini of Aβ38, Aβ40 and Aβ42 and were detected with SULFO-TAG™-labeled 6E10 antibody. Light emitted upon electrochemical stimulation was read using the MSD QuickPlex SQ120 instrument. Data were analyzed using MSD Workbench 4.0 software. Values of concentration in pg/mg of total protein were expressed as median and interquartil range (IQR). Mann Whitney test was applied to assess significant differences between groups.

**Measurement of NAD+ and NADH in rat hippocampal tissues.** NAD+/H levels were measured using NAD/NADH assay kit from Abcam (ab65348). Briefly, hippocampi from WT, Tg(+/-) and Tg(+/+) rats were snap frozen in liquid nitrogen, homogenized in NADH/NAD extraction buffer and filtered through a 10kD spin column (Abcam, ab93349) to remove enzymes. Assay procedure was followed per kit instructions and levels of NADH and NAD+ were determined normalized to tissue weight.

**Determination of enzymes transcript levels of NAD rate-limiting and NAD salvage pathway.** Transcript levels were estimated by reverse-transcription quantitative PCR (RT-qPCR). Protein, DNA and RNA were isolated from frozen hemi-hippocampus using Illustra™ TriplePrep Kit (GE Healthcare) following manufacturer’s instructions. Briefly, 1-3 ug of total RNA was reverse transcribed using oligo(dT) primer and SuperScript II reverse transcriptase (Invitrogen). Sequences of oligonucleotides (F, forward; R, reverse; 5′→3′) to assess transcript levels of NAD+ rate-limiting, NAD+ consumption, NAD+ synthesis enzymes and constitutive genes were as follows: Nicotinamide phosphoribosyltransferase (NAMPT) (F 5’-ATGCCGTGAAAAGAAGACAG-3’; R 5’-TCCAGTTGGTGAGCCAGTAG-3’); Cluster of differentiation 38 (CD38) (F 5’-GGTCCCTCAGTGAGCCATTT-3’; R 5’-ATGTCATGAATTACCCAGGC-3’), poli [ADP-ribosa] polimerase 1 (PARP1) (F 5’-AGGACCCCATCGATGTCAAC-3’; R 5’-GGTCGCGTGAGTGTTCTTCAC-3’), PARP2 (F 5’-ATGACGTCGTTCAAGCG-3’; R 5’-gtcatctgttgctctgttgcc-3’) Sirtuin 3 (SIRT3) (F 5’-TGTGGGGTCCGGGAGTATTA-3’; R-5’GTCATCTGTTGCTCTGTTGCC-3’); Nicotinamide Nucleotide Adenylyl transferase 2 (NMNAT2) (F 5’-TCCCAATATGACCGAGACCAC-3’; R 5’-TTGTGCAGATAATCCCTGGCT-3’);  Glyceraldehyde-3-phosphate dehydrogenase **(**GAPDH) (F 5’TACCCACGGCAAGTTCAA-3’; R 5’-ACCAGCATCACCCCATTT-3’) and Eukaryotic Translation Elongation Factor 1 Alpha 1 (EEF1A1) (F 5’-AACTGACAAGCCTCTGCGAC-3’; R 5’-GCTTCATGGTGCATTTCCACA-3’). RT-PCR reactions were prepared using PowerUp™SYBR™ Green Master Mix (ThermoFisher Scientific) and run following the standard cycling mode (primer Tm > 60°C) instructions in a Light Cycler 480 Instrument II (Roche). Melt curve analysis and agarose gels verified the presence of a single desired PCR product. Absolut quantification was performed using dilutions (1, 1:10, 1:50, 1:100, 1:500 and 1:1000) of a standard sample, which was a pool of 5 ul cDNA of each analyzed sample. The relative amount of transcripts to GAPDH or EEF1A1 was quantified by the 2^−ΔΔCt^ method using MxPro software. The mean ± SEM relative to WT (=1) were analyzed for each genotype and values above a fold-change of +1.5 were considered different from WT (=1).

**Supplementary Figures**

**
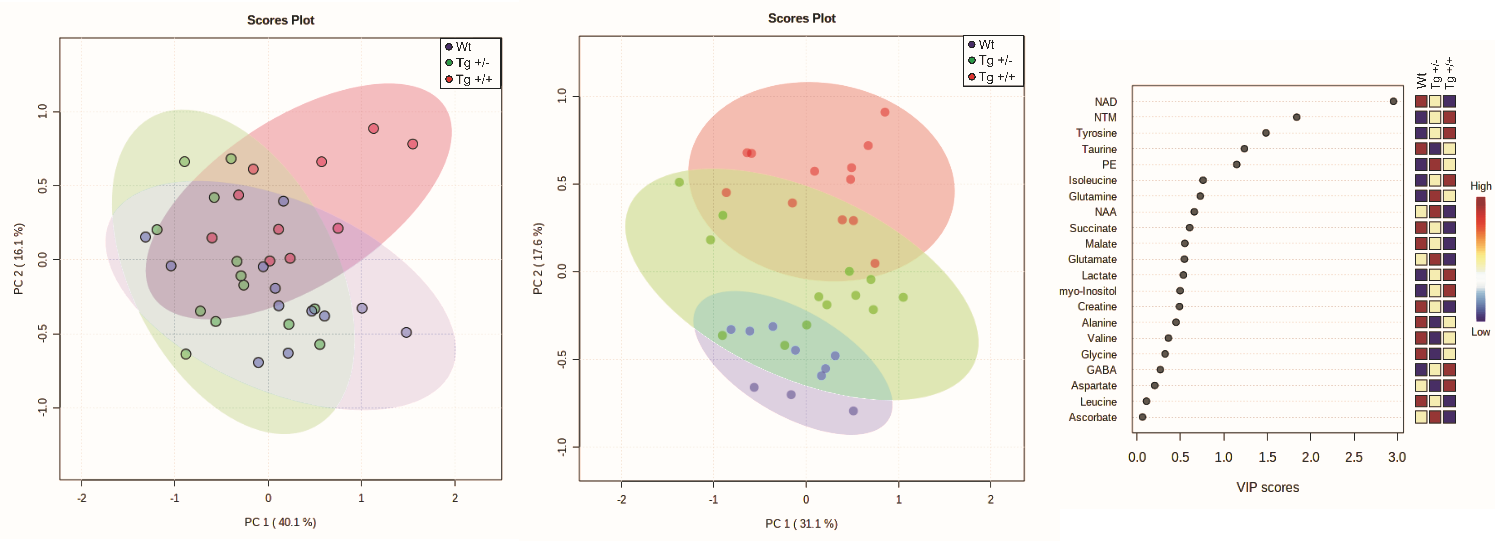
**

**Figure S1.** Principal component analysis (PCA) score plot, partial least square-discriminant analysis (PLS-DA) score plot and variable importance in projection (VIP) plot derived from the 600 MHz ^1^H NMR spectra of hippocampus samples. The predictive ability of the PLS-DA model was calculated via cross-validation (CV) (R2: 0.947 , Q2: 0.843) and validated by permutation testing (2000 iterations; P < 0.0005, unlikely to have occurred by chance). NAD, Nicotinamide adenine dinucleotide; NTM, Nicotinamide; PE, Phosphoethanolamine; NAA, N-acetylaspartate.


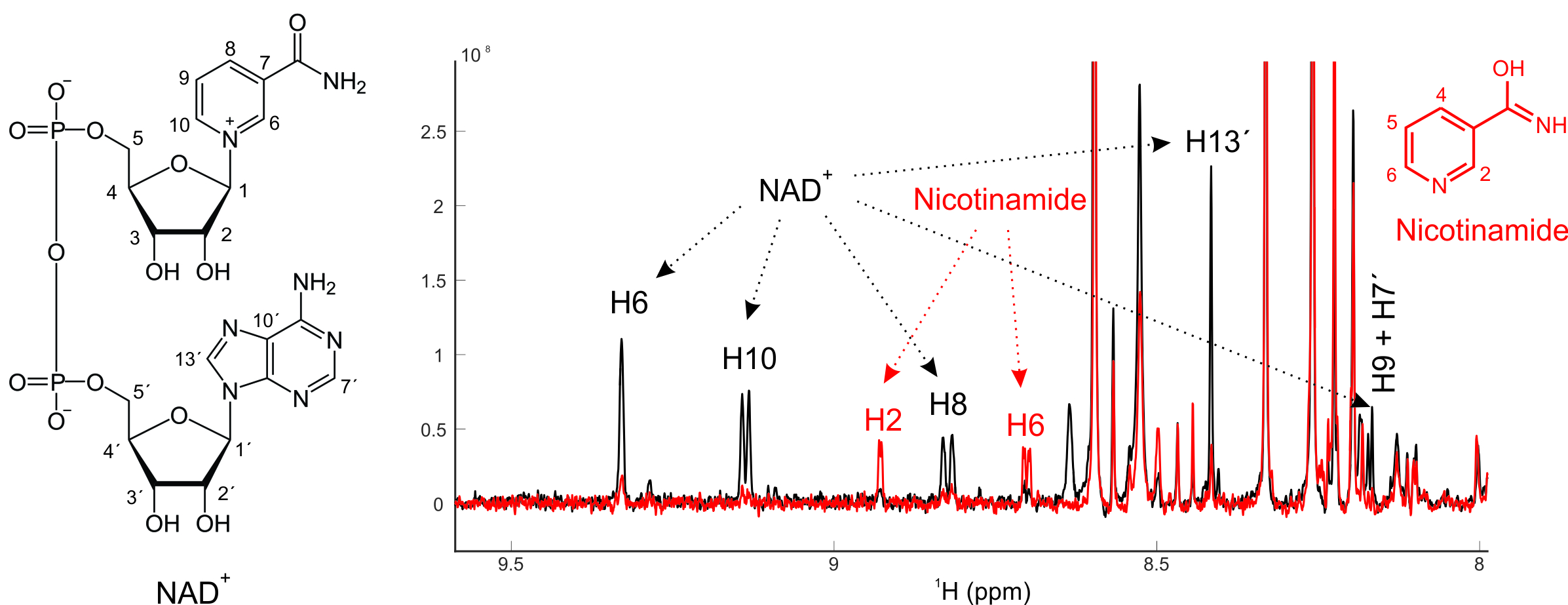


**Figure S2.** The 600-MHz ^1^H NMR NOESY spectra of a Wt rat (black) and a Tg +/+ rat (red) in the 9.5-8.0 ppm zone. The ^1^H resonances assigned to NAD^+^ and Nicotinamide are indicated and numbered according to the illustrated molecules.

**
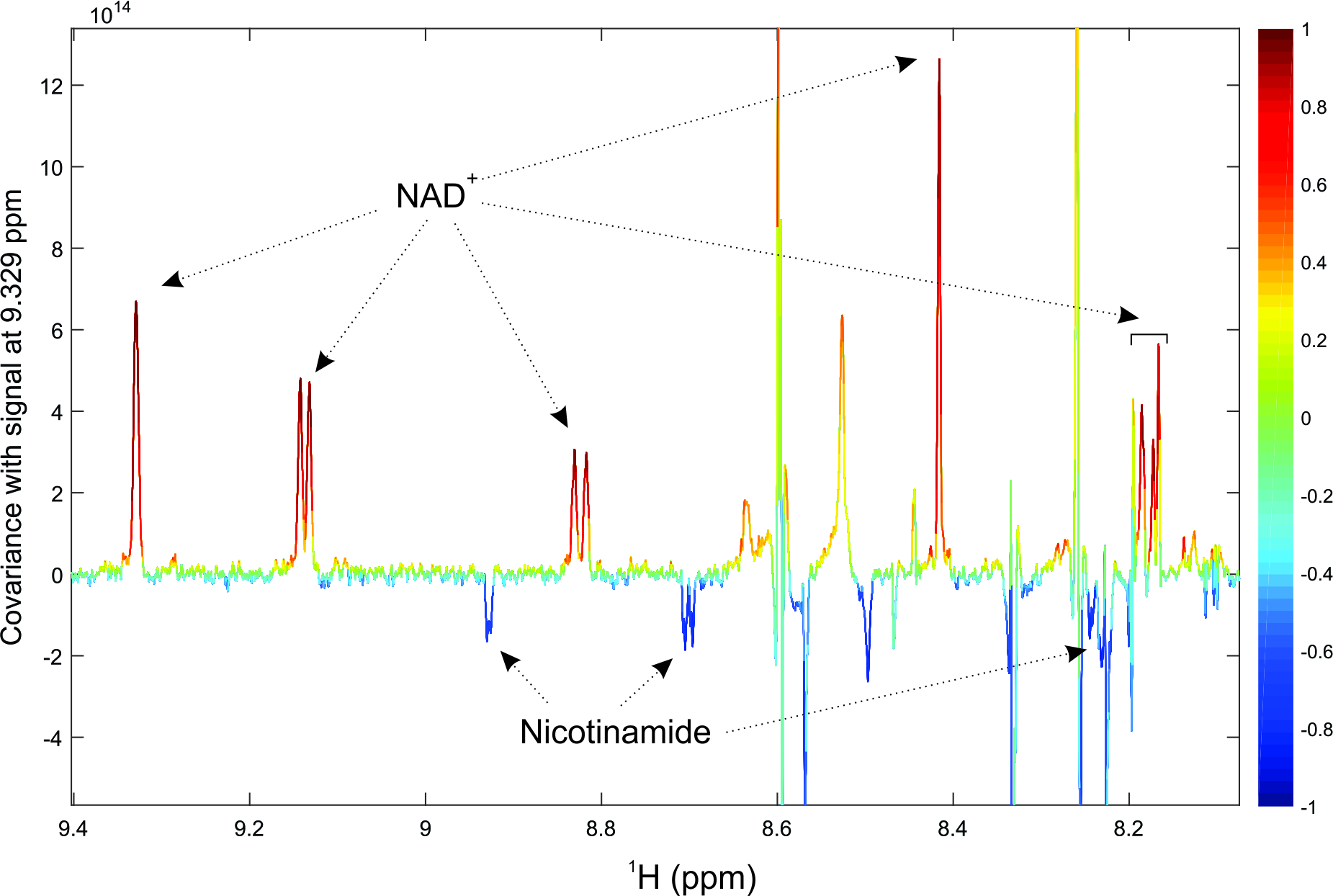
**

**Figure S3.** 1D STOCSY obtained using the CH (6) NAD^+^ peak at 9.329 ppm as the root peak, with color scale expressing the correlation (r) value. The ^1^H resonances assigned to NAD^+^ and Nicotinamide are indicated.

**Supplementary Table**

**Table S1.** NMR Resonance assignments of 27 metabolites from hyppocampus of experimental rats.

| **N°** | **Metabolites** | **Groups** | ** ^1^H (multiplicity)** | |  |
| --- | --- | --- | --- | --- | --- |
| 1 | **Alanine** | β-CH_3_ | 1,47 |  |  |
| 2 | **Acetic acid** | CH_3_ | 1,90 | (s) | |
| 3 | **Ascorbate** | β-CH | 4,50 | (m) | |
|  |  | γ-CH | 4,00 | (t) | |
|  |  | CH_2_ | 3,74 | (m) | |
| 4 | **Aspartate** | β-CH_2_ | 2,80 | (dd) | |
|  |  | α-CH | 3,89 | (dd) | |
| 5 | **Adenosine monophosphate (AMP)** | N-CH (ribose) | 6,13 | (d) | |
|  |  | CH (adenine) | 8,60 | (s) | |
|  |  | CH’ (adenine) | 8,26 | (s) | |
|  |  | OH-CH (ribose) | 4,50 | (dd) | |
|  |  | CH_2_-CH (ribose) | 4,38 | (dd) | |
| 6 | **Adenosine diphosphate (ADP)** | N-CH (ribose) | 6,13 | (d) | |
|  |  | CH (adenine) | 8,57 | (s) | |
|  |  | CH’ (adenine) | 8,47 | (s) | |
| 7 | **Adenosine-3´-monophosphate (3´AMP)** | N-CH (ribose) | 6,09 | (d) | |
|  |  | CH (adenine) | 8,33 | (s) | |
|  |  | CH’ (adenine) | 8,22 | (s) | |
|  |  | OH-CH (ribose) | 4,43 | (dd) | |
|  |  | CH_2_-CH (ribose) | 4,27 | (dd) | |
| 8 | **Creatine** | N-CH_3_ | 3,02 | (s) | |
|  |  | α-CH_2_ | 3,92 | (s) | |
| 9 | **γ-Aminobutyric acid (GABA)** | γ-CH_2_ | 3,00 | (t) | |
|  |  | β-CH_2_ | 1,98 | (m) | |
|  |  | α-CH_2_ | 2,28 | (t) | |
| 10 | **Glutamate** | γ-CH_2_ | 2,34 | (m) | |
|  |  | β-CH_2_ | 2,05 | (m) | |
|  |  | α-CH | 3,75 | (dd) | |
| 11 | **Glutamine** | γ-CH_2_ | 2,44 | (m) | |
|  |  | β-CH_2_ | 2,13 | (m) | |
| 12 | **Glycine** | N-CH_2_ | 3,55 | (s) | |
| 13 | **Glutathione (GSH)** | HN-C(=O)-CH_2_ | 2,55 | (m) | |
| 14 | **Isoleucine** | -CH_3_ | 0,99 | (d) | |
| 15 | **Lactate** | β-CH_3_ | 1,31 | (d) | |
|  |  | α-CH | 4,10 | (q) | |
| 16 | **Leucine** | -CH_3_ | 0,95 | (d) | |
|  |  | '-CH_3_ | 0,96 | (d) | |
| 17 | **Malate** | β-CH_2_ | 2,65 | (dd) | |
| 18 | **Methanol (res)** | CH_3_ | 3,34 | (s) | |
| 19 | **myo-Inositol** | -CH | 4,05 | (t) | |
|  |  | γ-CH | 3,52 | (dd) | |
|  |  | β-CH | 3,61 | (t) | |
|  |  | α-CH | 3,27 | (t) | |
| 20 | **N-acetylaspartate (NAA)** | CH_2_ (N-acetyl) | 2,01 | (s) | |
|  |  | β-CH_2_ | 2,68 | (dd) ; 2,48 (dd) | |
| 21 | **Nicotinamide adenine dinucleotide, oxidized (NAD^+^)*** | CH (6) | 9,33 | (s) | |
|  |  | CH (8) | 8,82 | (d) | |
|  |  | CH (10) | 9,14 | (d) | |
|  |  | CH (13´) | 8,42 | (s) | |
|  |  | CH (9) | 8,18 | (m) | |
|  |  | CH (7´) | 8,16 | (s) | |
| 22 | **Nicotinamide*** | CH (2) | 8,93 | (s) | |
|  |  | CH (6) | 8,70 | (dd) | |
| 23 | **Phosphoethanolamine (PE)** | N-CH_2_ | 3,25 | (t) | |
|  |  | O-CH_2_ | 4,05 | (m) | |
| 24 | **Succinate** | α-CH_2_ | 2,39 | (s) | |
| 25 | **Taurine** | N-CH_2_ | 3,25 | (t) | |
|  |  | S-CH_2_ | 3,41 | (t) | |
| 26 | **Tyrosine** | γ-CH, β-CH (phenyl) | 7,18 | (d); 6.89 (d) | |
| 27 | **Valine** | γ'-CH_3_ | 0,98 | (d) | |
|  |  | γ-CH_3_ | 1,03 | (d) | |
| Note: d, doublet；m, multiplet；q, quartet ； s, singlet；t, triplet; res: residual. *Atoms numbered according to Figure S2 | | | | | |
